# Prevalence of Non-Tuberculosis Mycobacterial Infections among Tuberculosis Suspects in Iran: Systematic Review and Meta-Analysis

**DOI:** 10.1371/journal.pone.0129073

**Published:** 2015-06-08

**Authors:** Mohammad Javad Nasiri, Hossein Dabiri, Davood Darban-Sarokhalil, Abdolrazagh Hashemi Shahraki

**Affiliations:** 1 Department of Microbiology, School of Medicine, Shahid Beheshti University of Medical Sciences, Tehran, Iran; 2 Department of Microbiology, School of Medicine, Iran University of Medical Sciences, Tehran, Iran; 3 Department of Epidemiology, Pasteur Institute of Iran, Tehran, Iran; Indian Institute of Technology Delhi, INDIA

## Abstract

**Introduction:**

The infections due to Non-Tuberculosis Mycobacteria (NTM) are becoming an important health problem in many countries in the world. Globally, an increase in NTM infections has been reported from many countries around the world. However, limited information is available about the prevalence of NTM infections in Iran.

**Material and Methods:**

The data of the prevalence of NTM infections were collected from databases such as PubMed, Web of science, Cochrane Library, Embase, Scopus, Iranmedex, and Scientific Information Database. Comprehensive Meta-Analysis (V2.0, Biostat) software was used to analyze the data.

**Results:**

The meta-analyses showed that the prevalence of NTM infections was 10.2% (95% confidence interval [95% CI] 6.3-15.9) among culture-positive cases of tuberculosis (TB) in Iran. The further stratified analyses indicated that the prevalence of NTM was higher in studies that were done after year 2000. Additionally, *M*. *simiae* (43.3% [95% CI 36.8-50.0]), *M*. *intracellucar* (27.3% [95% CI 0.7-95.5]) and *M*. *fortuitum* (22.7% [95% CI 16.1-30.9]) were the most prevalent NTM species, respectively.

**Discussion:**

The relatively high prevalence of NTM infections (10.2%) among culture positive cases for TB underlines the need for greater enforcement of infection control strategies. Establishment of appropriate diagnostic criteria and management guidelines for NTM diseases and expanding the number and quality of regional reference laboratories may facilitate more accurate action for prevention and control of NTM infections in Iran.

## Introduction

The infections due to Non-tuberculosis Mycobacteria (NTM) are an increasing problem in many countries in the world. NTMs are environmental organisms and are commonly found in soil, water, dust, animals and food. They are considered as opportunistic pathogens, and several species are capable of causing serious illnesses such as pulmonary disease, skin and soft tissue infection and disseminated infection in both immunocompetent and immunocompromised individuals [1, 2]. Based on reports from many developed countries, the numbers of diseases caused by NTMs are on the rise, and NTM accounts for an increasing proportion of mycobacterial disease [3–5]. Increase in the number of NTM associated diseases and patients may contribute to the implementation of developed molecular methods for detection of NTM and the growing population of patients susceptible to NTM infections (HIV cases). The rising number of NTM species is of concern as these are both difficult to diagnose and treat. Unfortunately, most of the NTM species are inherently resistant to the anti-tuberculosis (TB) agents, which make the treatment of these infections more difficult [6]. This concern is even more serious in economically challenged countries. In these regions, the prevalence of diseases caused by NTM is expected to rise due to inadequate laboratory facilities. In many cases identification of mycobacterium to the species level is not done and NTM diseases are frequently misdiagnosed as TB. Thus, this deferment in diagnosis of NTM can leads to development of variety of symptoms as well as high rate of morbidity and mortality [6]. In countries like Iran, where TB is still a major public health problem, the prevalence of NTM diseases among TB suspects has been rarely reported in literatures and a comprehensive analysis from different parts of Iran has not been performed yet. In the present study, we aimed to assess the exact magnitude of NTM infection and the diversity of microorganisms that found in Iranian population. Systematic review and meta-analysis according to the Preferred Reporting Items for Systematic reviews and Meta-Analyses (PRISMA) statement were used (S1 Table) [7].

## Methods

### Search Strategies

From January 1990 to September 2014, all studies addressing NTM infections in Iran were collected from databases PubMed, Web of Science, Embase, Scopus, Cochrane Library, Google Scholar, Iranmedex, and Scientific Information Database. The search was restricted to original research articles that have been published in English or Persian and present the prevalence or incidence of NTM infections in Iran. The following key words containing Medical Subject Headings or keywords in titles or abstracts were used with the help of Boolean operators (“and” or “or”): ‘‘non-tuberculosis”, ‘‘nontuberculosis”, ‘‘nontuberculous mycobacterium”, “non-tuberculosis mycobacterial”, ‘‘NTM”, ‘‘mycobacteria other than tuberculosis”, ‘‘MOTT”, ‘‘atypical mycobacterium” and ‘‘Iran”. Meanwhile, bibliographies from retrieved papers were investigated for any additional study. In addition to English papers, all relevant articles in Iranian databases such as Iranmedex (www.iranmedex.com), Scientific Information Database (www.sid.ir), Magiran (www.Magiran.com), Irandoc (www.irandoc.ac.ir) and Iranian National Library (www.nlai.ir) were searched with similar strategy and related Persian keywords.

### Inclusion and exclusion criteria

All original articles presenting cross-sectional or cohort studies on prevalence of NTM infections in Iran were considered. The selection of articles for review was done based on three stages: titles, abstracts, and full-text evaluation. The included studies should reference to the standard method for identification of NTM isolates. Standard methods include the conventional methods (i.e. niacin accumulation, growth in Lowenstein-Jensen (LJ) media containing thiophene-carboxylic acid hydrazide (TCH), growth at 42°C and 44°C, pigment production in light and dark, arylsulfatase activity, catalase, tween hydrolysis, nitrate reduction, tellurite reduction, tolerance to the NaCl 5% and urease) and molecular methods (e.g. PCR-RFLP and sequencing). In all included surveys, the effect size of the prevalence of NTM should be included in the studies. Studies were excluded from analysis for any of the following reasons: article has focused only on *Mycobacterium tuberculosis*; considered only specific groups such as HIV cases; considered only NTM cases co-infected with HIV and those that have not used standard methods. Review articles, meeting or congress abstracts that have been reported in languages other than English or Persian, meta-analyses or systematic reviews and articles available only in abstract form were also excluded.

### Data extraction and definitions

For all studies, the following data were extracted: the first author’s name, year of publication, year of study, study setting, number of cases investigated, method of studies, source of samples, sample size and prevalence of NTM infections. Two investigators extracted data from all of the included studies independently and results were reviewed by a third investigator. Inconsistencies between the reviewers were discussed to obtain consensus.

### Meta-Analysis

Analysis was performed by Comprehensive Meta-Analysis (V2.0, Biostat) software. The point estimates of effect size, the prevalence of NTM, and its 95% confidence interval (95% CI) were estimated for each study. Random effects models were used, taking into account the possibility of heterogeneity between studies, which was tested with the Cochran’s Q-statistic. In order to assess possible publication bias, Egger weighted regression methods were used. Value of P < 0.05 was considered indicative of statistically significant publication bias.

## Results

### Characteristics of Included Studies

**Fig 1:**
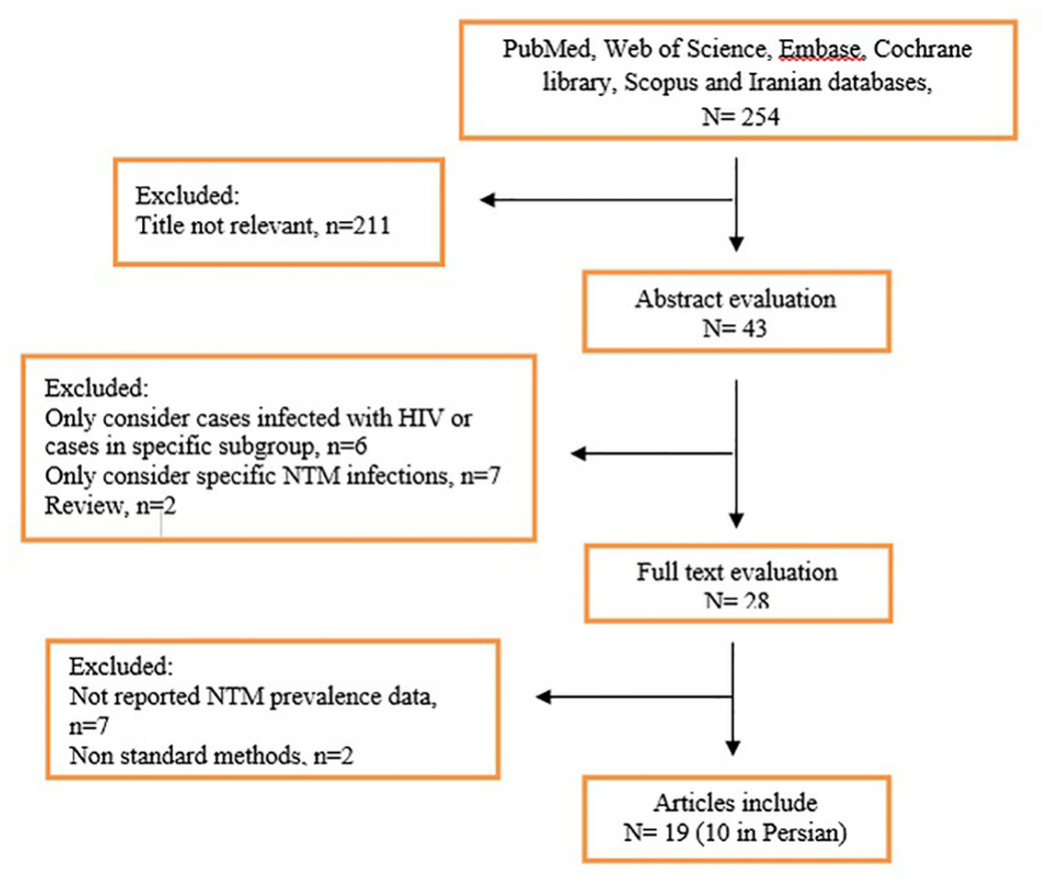
Flow diagram of literature search and study selection.

Initially, a total of 254 articles were collected (Fig 1). In secondary screening, 226 of them were excluded on the basis of the title and abstract evaluation. The exclusion of articles based on the title of papers was mainly because of the following reasons: the articles were based on case report, assessment of specific methods on NTM diagnosis, considered only specific groups of patients, reported NTM from environmental samples, reported NTM in specific disease, analysis of specific factor in non-tuberculosis infections. In the next step 9 of the remaining 28 studies were excluded upon a full text search; 7 have not reported NTM prevalence and 2 have used non-standard methods. Nineteen eligible studies were chose for further investigation. Characteristics of the selected articles are summarized in Table 1. Geographic location of studies covered east to west and north to south of Iran and the majority of patients were from central of Iran. Diagnostic methods for NTM mainly included conventional techniques. Additionally, NTM species were isolated from various clinical samples, including sputum, bronchoalveolar lavage (BAL), abscess, gastric washing, soft tissue infection, pleural samples, cerebrospinal fluid (CSF) and lymph node biopsy.

**Table 1:** Characteristics of studies included in the meta-analysis.

Study	Time of study	Published time	Province	N. of cases with suspected mycobacterial infections	N. of culture positive cases	N. of NTM isolates	Diagnostic methods for NTM
**Derakhshani** [14]	2003–2011	2014	Tehran	8322	4825	124	Conventional tests, PCR-RFLP
**Bahrmand** [15]	1993–1994	1996	Tehran	6472	525	82	Conventional tests
**Heidari** [16]	2007–2008	2009	Tehran	371	371	43	Conventional tests, PCR-RFLP
**Mohamadi** [17]	1996–1997	1998	Tehran	2272	186	30	Conventional tests
**Nasiri** [18]	2010–2012	2014	Tehran	6426	261	9	Conventional tests, sequencing
**Javid** [19]	2007–2008	2009	Golestan	-	104	17	Conventional tests
**Shafipour** [20]	2010–2011	2013	Golestan	3336	319	16	Conventional tests, sequencing
**Moghtaderi** [21]	2001–2010	2011	Tabriz	-	235	15	Conventional tests
**Heidarnejad** [22]	1999–2000	2001	Tabriz	165	165	10	Conventional tests
**Rohani** [23]	2007–2008	2009	Kashan	248	32	8	Conventional tests, PCR-RFLP
**Moniri** [24]	1999–2000	2001	Kashan	100	100	4	Conventional tests
**Nasrollahi** [25]	2010–2011	2012	Mazandaran	1345	65	6	Conventional tests, PCR-RFLP
**Namaei** [26]	2001–2002	2003	Mashhad	1700	98	8	Conventional tests
**Yazdi** [27]	2009–2010	2012	Yazd	32	32	1	Conventional tests
**Roayaei** [28]	1996–1997	1999	Khuzestan	6031	243	18	Conventional tests
**Hashemi** [29]	2009–2012	2013	Khuzestan	190	117	23	Conventional tests, sequencing
**Khosravi** [30]	2007–2008	2009	Khuzestan	150	88	8	Conventional tests, PCR-RFLP
**Farivar** [31]	2000–2004	2006	Sistan-Blochestan	150	150	59	Conventional tests
**Naderi** [32]	2003–2004	2006	Sistan-Blochestan	150	60	20	Conventional tests

### The Prevalence of NTM

**Fig 2:**
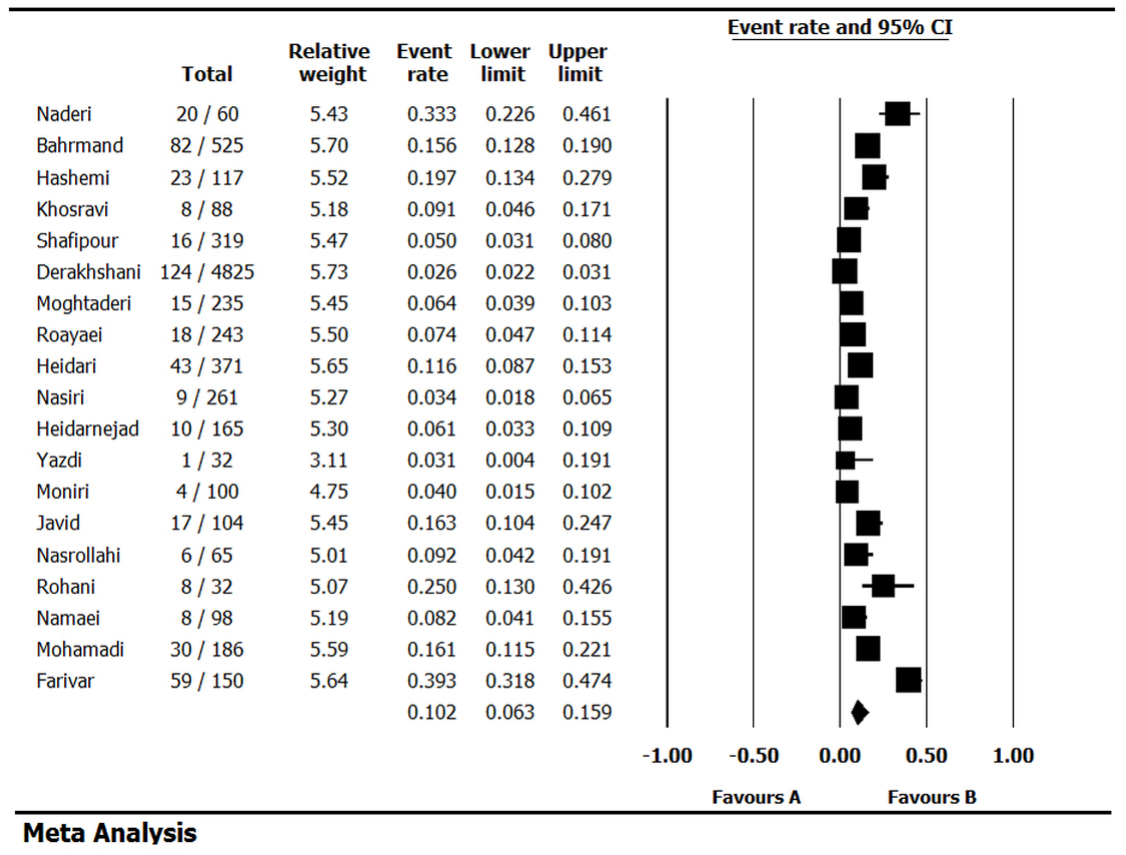
Forest plot of the meta-analysis on prevalence of NTM infections.

**Fig 3:**
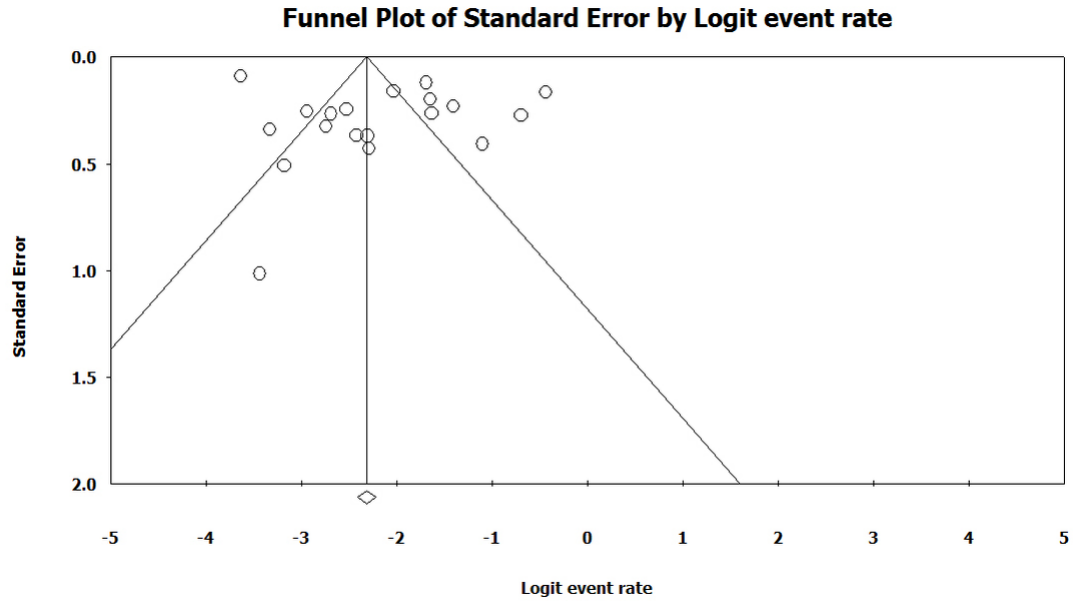
Funnel plot of the meta-analysis on prevalence of NTM infections.

The heterogeneity test indicated that there were heterogeneities between studies (*I*2 = 96.1, *p* < 0.001), so the random effect model was used to combine the prevalence of NTM. As it is shown in Table 2, the combined prevalence of NTM infections was 10.2% (95% CI 6.3–15.9) among culture-positive cases of TB in Iran. Fig 2 shows the forest plot of meta-analysis of NTM prevalence. Some evidence for publication bias was observed (Fig 3), however the results of Egger’s weighted regression test did not show bias in this study (*t* = 1.0, *p* > 0.05).

**Table 2:** Meta-analysis of prevalence of NTM infections in Iran.

Subgroups	No. of study	Prevalence of NTM (95% CI)	n/N	Heterogeneity test, I2 (%)	Heterogeneity test, P value	Egger’s test, t	Egger’s test, P value
**Overall effects**	19	10.2 (6.3–15.9)	501/7976	96.1	<.001	1.0	0.33
**Research before year 2000**	5	9.6 (6.0–15.0)	144/1219	83.4	<.001	3.2	0.04
**Research after year 2000**	14	10.6 (5.6–19.2)	357/6757	96.8	<.001	1.5	0.15

Abbreviations: n, number of events (NTM isolates); N, total number of culture positive cases.

### Prevalence of NTM species

As it is presented in Table 3, *M*. *simiae* (43.3% [95% CI 36.8–50.0]), *M*. *intracellucar* (27.3% [95% CI 0.7–95.5]) and *M*. *terrae* (18.3% [95% CI 11.3–28.2]) were the most prevalent NTM species among slowly growing mycobacteria (SGM) while *M*. *fortuitum* (22.7% [95% CI 16.1–30.9]), *M*. *abcsessus* (14.0% [95% CI 6.4–27.8]) and *M*. *chelonae* (7.6% [95% CI 2.8–18.8]) were the most prevalent NTM species among rapidly growing mycobacteria (RGM).

**Table 3:** Meta-analysis of species distribution among infected cases.

Classification	NTM species	No. of study	Prevalence of NTM (95% CI)	n/N	Heterogeneity test, I2 (%)	Heterogeneity test, P value	Egger’s test, t	Egger’s test, P value
**Slowly growing mycobacteria (SGM)**	*M*. *Kansassi*	7	13.1 (9.7–17.4)	40/316	0.0	0.70	0.6	0.54
	*M*. *simiae*	5	43.3 (36.8–50.0)	93/215	0.0	0.80	0.7	0.53
	*M*. *gordonae*	4	9.8 (3.6–24.2)	6/82	34.0	0.20	1.7	0.21
	*M*. *avium complex*	3	18.0 (6.7–40.5)	9/53	52.0	0.13	2.9	0.20
	*M*. *scrofulaceum*	3	6.1 (3.1–11.8)	8/143	0.0	0.43	1.4	0.39
	*M*. *szulgai*	3	8.6 (2.4–26.8)	7/115	65.8	0.05	0.6	0.63
	*M*. *gastri*	2	16.5 (5.0–42.7)	20/98	50.1	0.15	-	-
	*M*. *flavescens*	2	2.7 (0.5–12.8)	2/98	26.8	0.24	-	-
	*M*. *intracellucar*	2	27.3 (0.7–95.5)	8/51	92.9	0.00	-	-
	*M*. *nonchromogenicum*	1	6.3 (0.9–33.5)	1/16	-	-	-	-
	*M*. *terrae*	1	18.3 (11.3–28.2)	15/82	-	-	-	-
	*M*. *lentiflavum*	1	6.3 (0.9–33.5)	1/16	-	-	-	-
	*M*. *triviale*	1	5.6 (0.8–30.7)	1/18	-	-	-	-
	*M*. *asiaticum*	1	3.7 (1.2–10.7)	3/82	-	-	-	-
	*M*. *malmoense*	1	4.7 (1.2–16.8)	2/43	-	-	-	-
	*M*. *xenopi*	1	5.6 (0.8–30.7)	1/18	-	-	-	-
**Rapidly growing mycobacteria (RGM)**	*M*. *fortuitum*	6	22.7 (16.1–30.9)	43/197	22.3	0.26	1.1	0.30
	*M*. *chelonae*	4	7.6 (2.8–18.8)	27/265	69.4	0.02	2.5	0.12
	*M*. *fallax*	1	1.2 (0.2–8.1)	1/82	-	-	-	-
	*M*. *thermoresistibile*	1	1.2 (0.2–8.1)	1/82	-	-	-	-
	*M*. *smegmatis*	1	5.6 (0.8–30.7)	1/18	-	-	-	-
	*M*. *phlei*	1	4.9 (1.8–12.3)	4/82	-	-	-	-
	*M*. *abcsessus*	1	14.0 (6.4–27.8)	6/43	-	-	-	-

Abbreviations: n, number of events (NTM isolates); N, total number of culture positive cases.

## Discussion

The current systematic review reports the prevalence of NTM infections in Iran and examines the most prevalent NTM species among infected cases. The overall meta-analysis indicated that the prevalence of NTM infections was 10.2% (95% CI 6.3–15.9) among culture-positive cases of TB (Table 2). The analysis also showed that the pool prevalence of NTM infections was higher when the time of study was after year 2000 (from 9.6% in studies before 2000 to 10.6% in studies after 2000). The reasons for the increased reports of NTM infections may be related to: recognition of the importance of disease by physicians and microbiologists; improvement of laboratory facilities; and increasing the prevalence of immunocompromised hosts that causes increase in NTM infection in the general population [8]. Overall, the relatively high prevalence of NTM infections (10.2%) in Iran may have several negative effects on public health. First of all, the clinical manifestations of NTM infections are frequently overlapped with diseases caused by *M*. *tuberculosis* that makes the specific diagnosis of NTM difficult [9, 10]. Second, the increasing cases of NTM can yield false positive results in direct sputum-smear microscopy of acid-fast bacilli (AFB) which can pose a challenge for a directly observed treatment–short course (DOTS) program. Under the DOTS strategy, treatment of TB is started only on the basis of sputum microscopy results [9]. Therefore, a false-positive AFB can put NTM infected individuals on anti-TB chemotherapy, even though the treatment of NTM disease is not similar to treatment of TB [6, 9]. Third, the most NTM infections typically occur only during severe immune suppression when the T lymphocyte number falls below the baseline level. Therefore, HIV/AIDS patients are significantly more vulnerable to NTM infections due to their suppressed immune system. It is important in studied country where it is estimated that more than 20 thousands of people are infected with HIV virus [11]. Moreover, the standardized or accepted criteria to define NTM disease are missing in Iran that makes the diagnosis more difficult.

Another important finding of this study was the high prevalence of RGM such as *M*. *fortuitum*, *M*. *abcsessus* and *M*. *chelonae* (Table 3). These species are the most common NTM isolates associated with nosocomial disease [12]. Previous studies demonstrated that tap water, ice prepared from tap water, processed tap water used for dialysis, and piped water systems in hospital settings are the usual nosocomial sources of NTM infections [12]. Compare to *M*. *tuberculosis*, NTMs are even more difficult to eradicate with common decontamination practices and are relatively resistant to standard disinfectants such as chlorine and alkaline glutaraldehydes [13]. Furthermore, each NTM species may have its own antibiotic susceptibility pattern and they are usually more resistance to antimicrobial agents compared to *M*. *tuberculosis* and SGM [6]. In this regards, transmission and spread of these NTM species from nosocomial sources may constitute the major part of the problem in hospital control strategies.

Some limitations of this review should be discussed. First of all, only published studies were included in the current meta-analysis. Thus, as with any systematic review, existence of potential publication bias should be considered. Second, heterogeneity was detected among the included studies. Third, it cannot fully represent the prevalence of NTM infections in Iran because the extent of NTM infections is not yet examined in many areas of the country. In conclusion, the increase in the number of NTM infections is a serious public health problem in Iran and merit further attention by health authorities, physicians, and microbiologists. Establishment of appropriate diagnostic criteria and management guidelines for NTM diseases and expanding the number and quality of regional reference laboratories may facilitate more accurate action for prevention and control of NTM infections in Iran. The recognition of NTM as an ‘emerging pathogens’ would perhaps elevate the status of NTM in infections control strategies.

## Supporting Information

S1 Table PRISMA Checklist. (DOC)
